# Proteomics of regenerated tissue in response to a titanium implant with a bioactive surface in a rat tibial defect model

**DOI:** 10.1038/s41598-020-75527-2

**Published:** 2020-10-28

**Authors:** Raluca M. Boteanu, Viorel I. Suica, Luminita Ivan, Florentina Safciuc, Elena Uyy, Emanuel Dragan, Sorin M. Croitoru, Valentina Grumezescu, Marioara Chiritoiu, Livia E. Sima, Constantin Vlagioiu, Gabriel Socol, Felicia Antohe

**Affiliations:** 1 Institute of Cellular Biology and Pathology “N. Simionescu” of the Romanian Academy, 8, B.P. Hasdeu Street, PO Box 35-14, 050568 Bucharest, Romania; 2 Faculty of Engineering and Management of Technological Systems, Politehnica University of Bucharest, Bucharest, Romania; 3 National Institute for Lasers, Plasma and Radiation Physics, 409 Atomistilor Street, Magurele, P.O. Box MG-54, 77125 Bucharest, Romania; 4 Institute of Biochemistry of the Romanian Academy, Bucharest, Romania; 5 Faculty of Veterinary Medicine, University of Agronomic Sciences and Veterinary Medicine of Bucharest, Bucharest, Romania

**Keywords:** Bioinformatics, Biological models, Immunological techniques, Mass spectrometry, Microscopy, Proteomic analysis, Fracture repair

## Abstract

Due to their excellent mechanical and biocompatibility properties, titanium-based implants are successfully used as biomedical devices. However, when new bone formation fails for different reasons, impaired fracture healing becomes a clinical problem and affects the patient's quality of life. We aimed to design a new bioactive surface of titanium implants with a synergetic PEG biopolymer-based composition for gradual delivery of growth factors (FGF2, VEGF, and BMP4) during bone healing. The optimal architecture of non-cytotoxic polymeric coatings deposited by dip coating under controlled parameters was assessed both in cultured cells and in a rat tibial defect model (100% viability). Notably, the titanium adsorbed polymer matrix induced an improved healing process when compared with the individual action of each biomolecules. High-performance mass spectrometry analysis demonstrated that recovery after a traumatic event is governed by specific differentially regulated proteins, acting in a coordinated response to the external stimulus. Predicted protein interactions shown by STRING analysis were well organized in hub-based networks related with response to chemical, wound healing and response to stress pathways. The proposed functional polymer coatings of the titanium implants demonstrated the significant improvement of bone healing process after injury.

## Introduction

Titanium and some of its alloys are bioinert materials used in orthopedic implants that replace hard tissue due to their excellent mechanical and biocompatibility properties. However, new bone formation may fail for different reasons, resulting in delayed unions or non-union fractures^1^. Impaired fracture healing represents a clinical problem that affects the patient’s quality of life. The treatment can be also difficult, time-consuming, and costly^2^. To overcome these drawbacks, implants designed to carry and deliver bioactive molecules capable of enhancing the cellular processes that boost the bone repair and regeneration present a good treatment alternative^1,3^. Previous reports show a rapid bone healing for different in vivo models after embedding of growth factors such as fibroblast growth factor-2 (FGF2), vascular endothelial growth factor (VEGF) or bone morphogenetic proteins (BMPs) in different polymeric hosts^4–7^. Two applications of treatments with human recombinant BMPs (rhBMP-2 and rhBMP-7) have already been approved for limited clinical use^8^, since several adverse effects have been described^9–12^.

The purpose of this study was to develop a 3D hierarchical biomaterial structure by dip coating method that would gradually release FGF2 and VEGF before BMP-4 osteogenic factor deposited in a subjacent polymeric scaffold, to improve bone healing. BMP4 was identified to be present during all stages of bone development and regeneration^13^. FGF2 is an essential growth factor in tissue repair and bone regeneration^14^ acting as a stem cell self-renewal stimulating factor^15^ and specifically, in the case of mesenchymal stem cells, maintaining skeletal precursors in a pro-endochondral ossification state^16^. Consequently, FGF2 prevents spontaneous uncontrolled commitment to other fates (e.g. adipogenic) before activation of osteogenic BMP-driven differentiation signaling. In addition, VEGF was added in this experiment to ensure proper tissue neo-vascularization that would favor new bone tissue formation. Besides controlling endothelial cell activities, VEGF is a direct modulator of bone development^17^ by stimulating differentiation of periosteal progenitor cells to osteoblasts^18^ and regulating osteoclastic differentiation and migration^19^. Despite of all this knowledge related to bone regeneration assisted by titanium implant, detailed mechanisms driving proper bone healing are insufficiently understood, requesting further in-depth studies.

In this study, using biochemical and proteomics analyses, we developed and evaluated an implant of titanium coated with a Poly (ethylene glycol), (PEG) matrix containing FGF2, VEGF and Poly (3-hydroxybutyrate-co-3-hydroxyvalerate), (PHBV) microspheres embedded with BMP4 in a rat diaphyseal in vivo tibial defect model. The regenerative potential of the surgical inserted implants was closely monitored to identify downstream protein effectors involved in wound healing. The histological and histomorphometric data demonstrated that the implant incorporating all three growth factors accelerates the healing of bone defect in comparison with the other examined implants that had similar growth factors, individually included or in binary combinations. In addition, a set of common and unique over or under expressed proteins were identified in all groups at the site of bone defect using mass spectrometry analysis. Based on Pearson correlation matrices between these proteins and using bioinformatics analysis, a novel distinct protein network associated with several gene ontology (GO) pathways such as *response to chemical*, *wound healing* and *response to stress* was generated for each group. The reported results reveal the biological effects of the interaction between titanium implants with bioactive surface and bone tissue. Moreover, the new research uncovered by mass spectrometry provides an extended list of proteins that may open up better bone wound treatment strategies in the future.

## Results

### In vitro evaluation of the biocompatibility of the implants with functionalized surface

**Figure 1:**
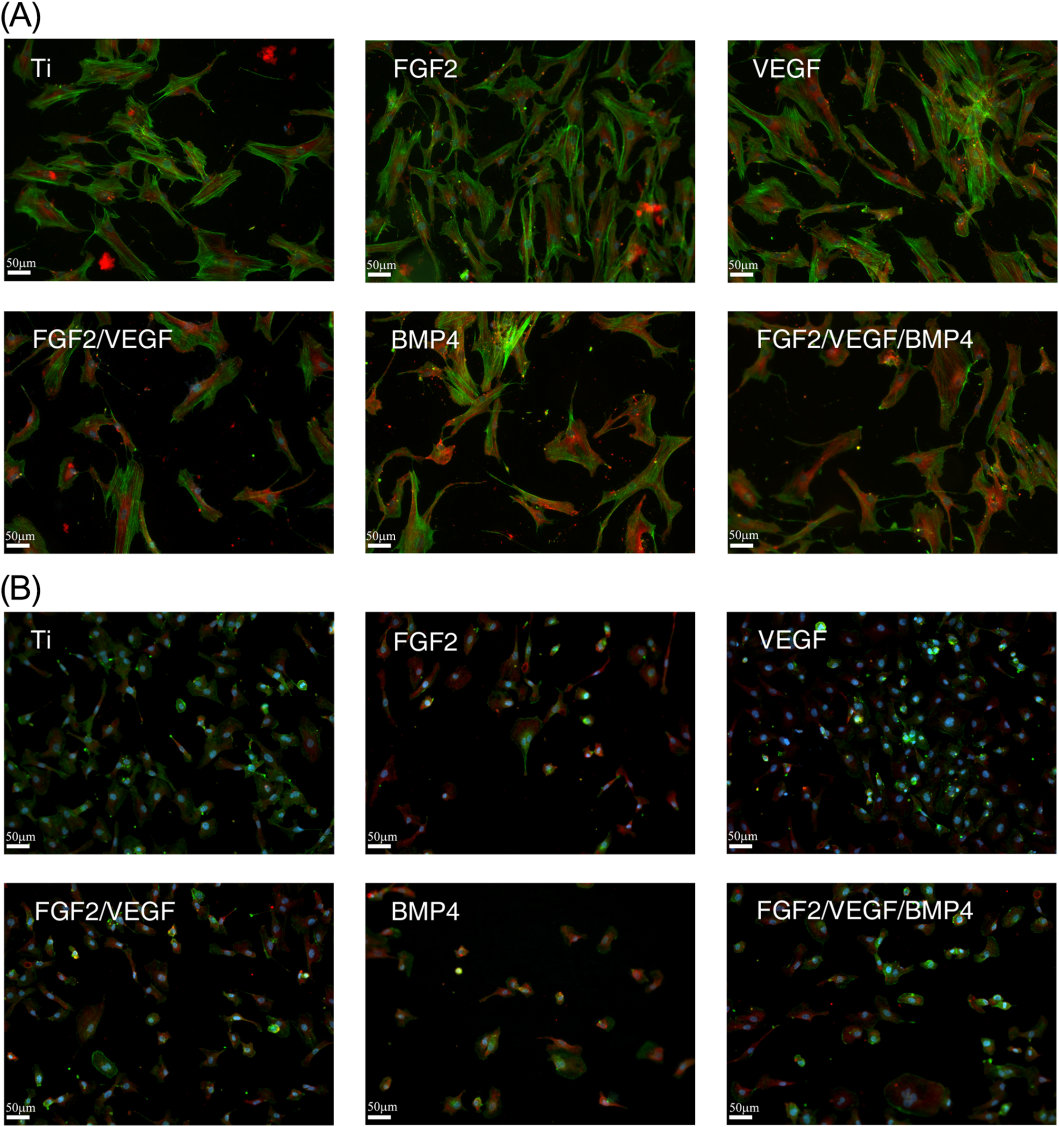
Adhesion of human mesenchymal stem cells (**A**) and endothelial cells (**B**) onto the tested biomaterials. Immunofluorescent staining for actin filaments (green), vinculin (red) and nuclei (blue) was performed at 72 h post-seeding. Data represent one of three performed independent experiments. *Ti* titanium, *FGF2* fibroblast growth factor 2, *VEGF* vascular endothelial cell growth factor, *BMP4* bone marrow protein 4.

The main cell types involved in bone tissue regeneration are the mesenchymal stem cells (MSCs) and the endothelial cells (ECs). While the MSCs represent the source of osteogenic progenitor pool, ECs forming the blood vessels provide nutrients and signals necessary for bone repair. Therefore, we tested the effect of the proposed biomaterials on the adhesion and proliferation of human MSCs (hMSCs) and ECs. Immunofluorescence experiments staining for actin filaments and vinculin showed that all titanium coatings supported cell adhesion (Fig. 1).

Moreover, FGF2 increased hMSCs coverage (Fig. 1A) and VEGF enhanced ECs' observed density (Fig. 1B) at 72 h post-seeding, as compared to titanium control and the polymeric films containing other growth factors. These results are consistent with the proliferation assay (see Supplementary Fig. S1 online).

### In vivo and ex vivo evaluation of bone healing

**Figure 2:**
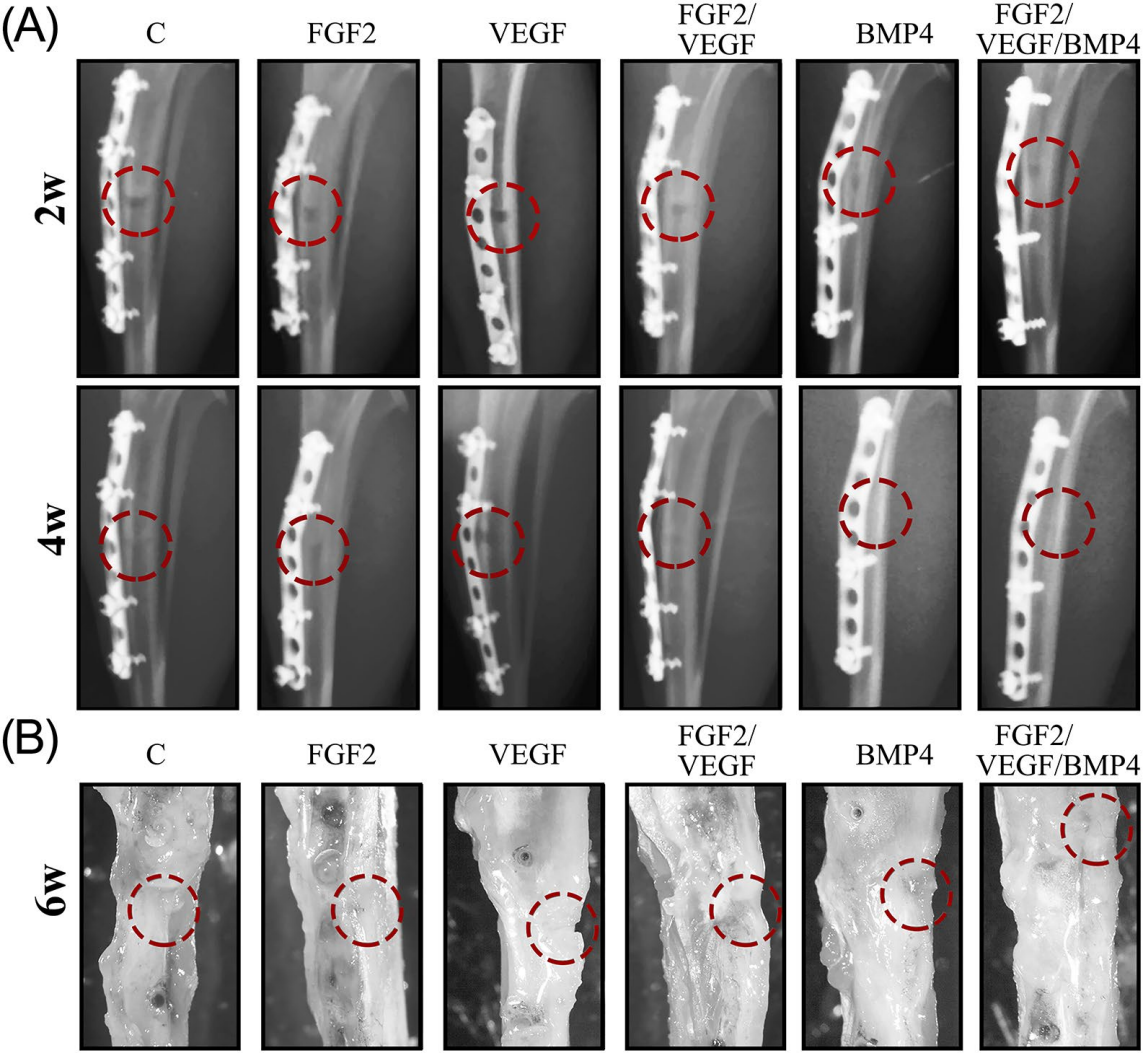
Evolution of bone repair during the 6 weeks experimental period. (**A**) Representative radiographs of defect site of control C, FGF2, VEGF, FGF/VEGF, BMP4 and FGF/VEGF/ BMP4 groups, at 2 (2w) and 4 (4w) weeks after surgery. There were no differences among the groups regarding the healing of bone defect at 2 weeks. X-ray images at 4 weeks showed a less visible defect for BMP4 and FGF/VEGF/ BMP4 groups suggesting an accelerated repair process compared to the other groups. (**B**) At 6 weeks (6w) after implantation, the tibiae were evaluated using the stereomicroscope. The images (× 0.65 magnification) indicated a very good healing with hard to distinguish edges between the newly formed and mature bone only in the FGF2/ VEGF/ BMP4 group. The red circles indicate the bone defect areas.

After validating titanium coatings biocompatibility in vitro*,* we next pursued testing of implants’ healing capacity in vivo in a rat animal model of tibia injury (see Supplementary Fig. S2 online). X-ray images at 2 weeks after surgery showed the presence of the bone defect in all investigated groups with no prominent differences among them (Fig. 2A). At 4 weeks, the bone defect persisted in the animals with implants covered with FGF2 and VEGF as revealed by the radiographs. The control and the FGF2/ VEGF groups presented a less visible defect. Oppositely, both BMP4 and FGF2/ VEGF/ BMP4 groups unveiled a defect zone filled with hard callus suggesting that the new bone presented increased mineralization (Figs. 2A, 4W). Stereomicroscope examination of the ex vivo tibiae showed the presence of the bone defect in all groups. However, it was difficult to differentiate between the edge of newly formed bone and mature bone for the FGF2/ VEGF/ BMP4 group (Fig. 2B, last panel) while the exception animal from VEGF group had a defect not completely filled with newly formed tissue at 6 weeks after operation (Fig. 2B, 3rd panel). These results suggest an essential role for BMP4 in enhancing the bone defect repair in our rat model.

### Histology of bone repair

**Figure 3:**
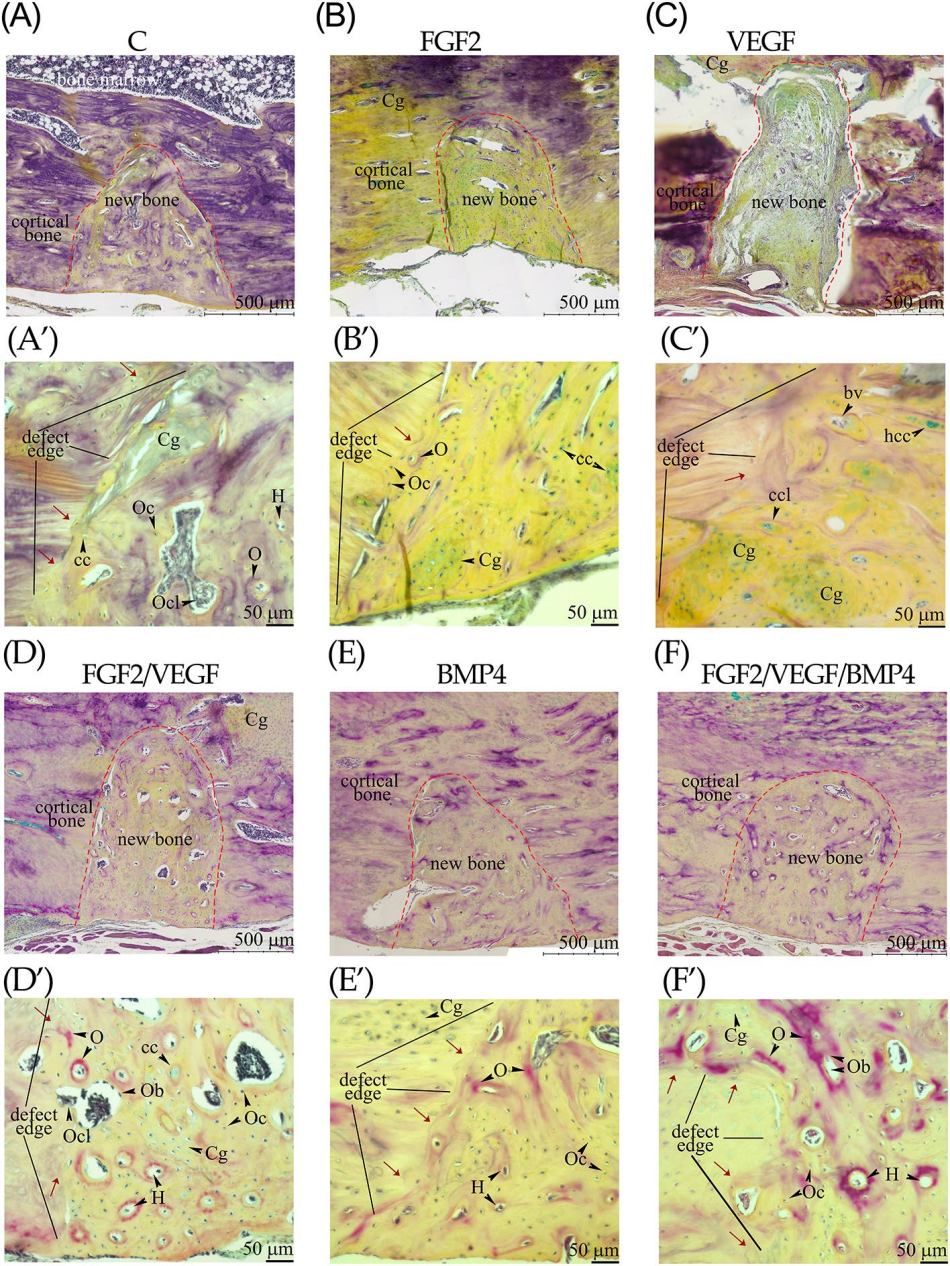
Representative histological images of tissues harvested from the bone-implant interface. The bone defect sites of the control (**A**,**A’**), FGF2 (**B**,**B’**), VEGF (**C**,**C’**), FGF/VEGF (**D**,**D’**), BMP4 (**E**,**E’**) and FGF/VEGF/ BMP4 (**F**,**F’**) groups at 6 weeks post-surgery are shown at two different magnification. Decalcified longitudinal sections (Movat’s pentachrome staining) are oriented with the bone defect on the bottom in all micrographs. The red dotted line points out the edge between the defect and the compact bone (**A**–**F**). The black lines indicate the defect margins (**A’**–**F’**). Scale bar: 500 μm (**A**–**F**) and 50 μm (**A’**–**F'**). *Cg* cartilage, *cc* chondrocyte, *ccl* chondroclast, *Oc* osteocyte, *Ob* osteoblast, *Ocl* osteoclast, *H* Haversian canal, *O* osteoids, *bv* blood vessel, *rl* resorption lacuna.

Further, to detect whether the investigated implants could accelerate bone repair, the morphology of the newly formed bone was examined on histology sections stained with Movat’s pentachrome. In all groups, new bone appears to be incorporated or attached to the mature cortical bone of tibia suggesting a complete bone healing (Fig. 3A,B,D,E,F) except the animal from VEGF group (Fig. 3C). Distal from the bone defect, large areas of cartilage were present in FGF2, VEGF and FGF2/VEGF implanted animals (Fig. 3B–D). In the woven bone of the control group cartilage tissue and chondrocytes, disorganized collagen fibers, osteoclasts (in resorption lacunae), osteocytes, Haversian units, and osteoids were identified (Fig. 3A’) and some remodeling regions became visible near the defect edge (Fig. 3A’, red arrows). FGF2 group showed a maturing bone tissue characterized by numerous chondrocytes and hypertrophic chondrocytes (Fig. 3B), consistent with previous reports^20,21^. There were few osteoids and mature osteocytes embedded in lacunae located at the defect edge where the bone remodeling was initiated (Fig. 3B’). The formation of new bone tissue in the VEGF animals showed variable phases of bone healing at 6 weeks after surgery. One animal did not exceed the soft callus stage in which fibrocartilage tissue and blood vessels prevailed (Fig. 3C and see Supplementary Fig. S3 online). A second VEGF animal had large areas of cartilage within the defect (Fig. 3C’ and see Supplementary Fig. S3 online) and for the last one, the woven bone presented many osteoids and was highly remodeled (see Supplementary Fig. S3 online). FGF2/VEGF group presented numerous resorption lacunae in the area of newly formed bone with osteoblasts and osteoclasts and many Haversian canals as compared to control (Fig. 3D’).

Evaluation of the regenerated bone in both BMP4 and FGF2/VEGF/BMP4 groups showed a less visible cartilage tissue, an increased number of osteocytes and the remodeling of the woven bone was advanced along the defect margins than in the control (Fig. 3E’ and 3F’). In addition, organized collagen fibers were only detectable in FGF2/VEGF/BMP4 group (Fig. 3F’).

### Histomorphometry of the bone defects areas

**Figure 4:**
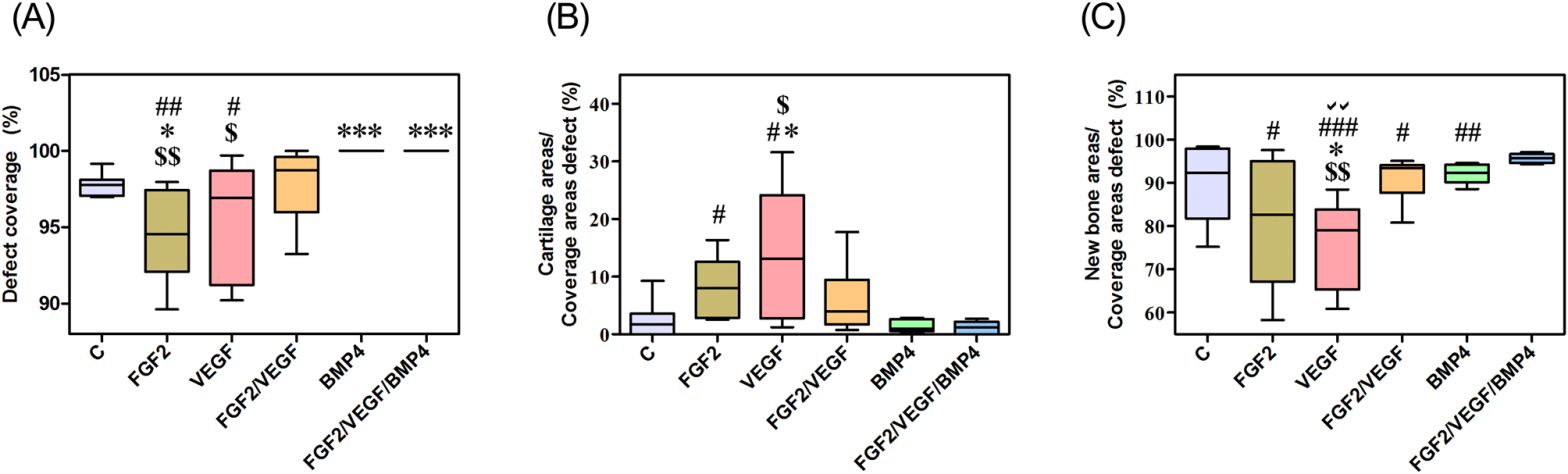
Histomorphometric analysis of bone regeneration at 6 weeks after implantation was performed by using the ImageJ software version 1.50i. (**A**) The results reported as percentage of total area show that the defect coverage was complete for BMP4 and FGF2/ VEGF/ BMP4 groups. (**B**) The ratio of cartilage tissue to coverage defect was significantly increased in the VEGF group compared to control C, BMP4 and FGF2/ VEGF/ BMP4 groups. (**C**) The amount of new bone area reported to coverage defect was higher in the FGF2/ VEGF/ BMP4 group compared to the other conditions. Unpaired 2-tailed Student’s t test was used. *p < 0.05, ***p < 0.001 vs C; ^$^p < 0.05, ^$$^p < 0.01 vs BMP4; ^#^p < 0.05, ^##^p < 0.01; ^###^p < 0.001 vs FGF2/ VEGF/ BMP4; √√p < 0.01vs FGF2/ VEGF.

Histomorphometric analysis established that the defect coverage was complete in the case of both BMP4 and FGF2/VEGF/BMP4 groups as opposed to the control (p < 0.001), FGF2 (p < 0.01) and VEGF (p < 0.05) samples as shown in Fig. 4A.

A statistically significant increase (p < 0.05) of the cartilage areas (13.98 ± 4.68) was observed for VEGF group compared to the control (2.57 ± 1.20), BMP4 (1.366 ± 0.43) and FGF2/VEGF/BMP4 (1.19 ± 0.44). The cartilage areas were also significantly increased in the FGF2 group (7.74 ± 2.48; p < 0.05) as compared to the FGF2/ VEGF/ BMP4 group (Fig. 4B). The percentage of the new bone area reported to the coverage defect was significantly higher in the FGF2/ VEGF/ BMP4 group compared to the other experimental conditions excepting the control (Fig. 4C). Even if the difference between the two groups was not significant, still the new bone area was higher in the FGF2/ VEGF/ BMP4 group (95.66 ± 0.43) than in the control (89.98 ± 3.70).

### Proteomics of the regenerated tissue located at the implant: bone interface

**Figure 5:**
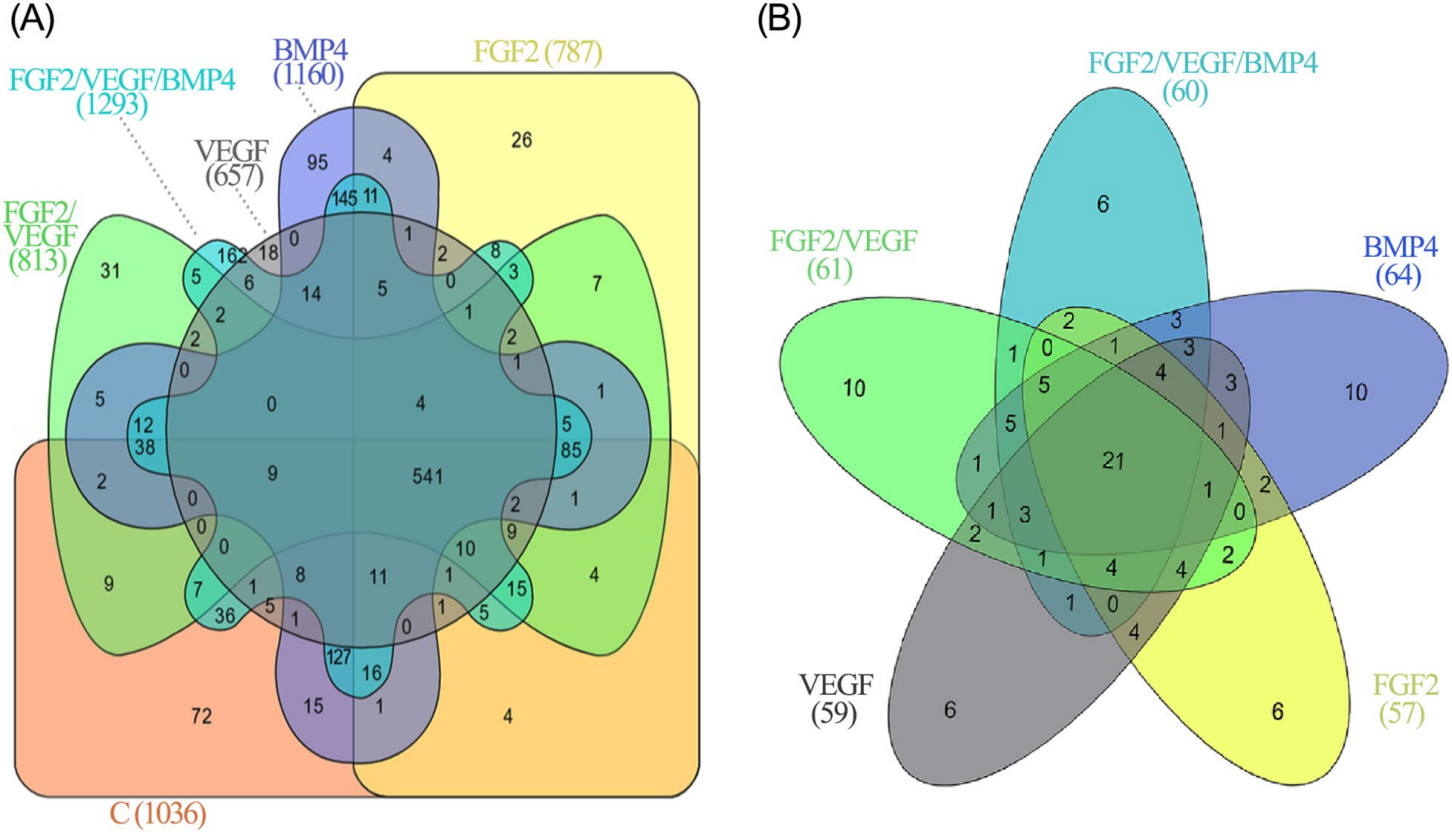
(**A**) Venn diagram showing the distribution of the total proteins identified (1614) in at least one biological replicate from each group of control C: 1036 (orange), FGF2: 787 (yellow), VEGF: 657 (grey), FGF2/VEGF: 813 (green), BMP4: 1160 (blue) and FGF2/VEGF/BMP4: 1293 (light blue) as well as the unique and the overlapping proteins among the groups. (**B**) Venn analysis performed for a total of 113 proteins differentially expressed in the experimental groups evidenced 21 proteins that are overlapped in all groups. Supporting Information shows the lists of differentially expressed proteins (Table S1).

To examine the proteome of fragments harvested from the site of bone healing under the influence of investigated implants, we applied high-performance LC–MS/MS-based proteomic analysis. The shotgun proteomic approach allowed the high-confidence identification of 1614 proteins in total, out of which 541 proteins were common to all groups (Fig. 5A).

BMP4 and FGF2/ VEGF/ BMP4 groups had a considerable overlap of 145 proteins whereas 127 proteins represented a distinctive signature of the intersection between control, BMP4 and FGF2/ VEGF/ BMP4 groups that corroborate well with the histomorphometric evaluation. On the contrary, the overlap of VEGF, FGF2/VEGF, BMP4 and FGF2/VEGF/BMP4 groups showed no identified proteins. Unique proteins were present in each group (Fig. 5A) with the mention that FGF2/ VEGF/ BMP4 group was at the upper extremity with the highest percentage of uniquely identified proteins (12.5%) whereas VEGF group was at the lower extremity (2.7%) followed closely by FGF2 (3.3%) and FGF2/VEGF (3.8%) groups. The distribution of the identified proteins corresponding to GO slim analysis is given in Supplementary Fig. S4 online.

Further, we focused on differentially expressed proteins. In all investigated groups, a total of 113 differentially expressed proteins (listed in Supplementary Table S1 online) were detected, including 21 common proteins and those uniquely attributed to each group (Fig. 5B)*.* Using GO slim analysis, these proteins were classified according to *biological processes* as shown in Supplementary Fig. S5 online. Therefore, *response to stimulus, regulation of biological process* and *metabolic process* were the classes that gathered the majority of differentially expressed proteins. The high proportion of the common differentially expressed proteins (17 out of 21) was clustered in the *response to stimulus* class. The unique differentially expressed proteins evidenced in the same category are listed in Table 1.

**Table 1:** List of unique differentially expressed proteins gathered in the *response to stimulus* class of GO slim biological processes with normalized abundance ratio (of each group versus control).

Group	Gene symbol	Protein names	Ratio (vs C)	St dev	P-value
FGF2	UQCRC1	Cytochrome b-c1 complex subunit 1, mitochondrial	0.465	0.045	p < 0.001
	GPD1	Glycerol-3-phosphate dehydrogenase [NAD( +)], cytoplasmic	0.597	0.037	p < 0.001
	PRDX2	Peroxiredoxin-2	1.942	0.173	p < 0.001
	CFL1	Cofilin-1	2.303	0.176	p < 0.001
VEGF	HNRNPK	Heterogeneous nuclear ribonucleoprotein K	0.277	0.022	p < 0.001
	APOE	Apolipoprotein E	0.164	0.021	p < 0.001
	THBS4	Thrombospondin-4	2.449	0.341	p < 0.001
FGF2/VEGF	EEF2	Elongation factor 2	0.526	0.021	p < 0.001
	PSAP	Prosaposin	1.694	0.054	p < 0.001
	GOT1	Aspartate aminotransferase, cytoplasmic	0.399	0.033	p < 0.001
	IGH-1A	Ig gamma-2b chain C region	0.386	0.07	p < 0.001
	EEF1A2	Elongation factor 1-alpha 2	0.356	0.02	p < 0.001
BMP4	APOA1	Apolipoprotein A-I	0.351	0.091	p < 0.001
	PEBP	Phosphatidylethanolamine-binding protein 1	0.293	0.04	p < 0.001
	HSPA8	Heat shock cognate 71 kDa protein	0.306	0.03	p < 0.001
	HSPD1	60 kDa heat shock protein, mitochondria	0.300	0.038	p < 0.001
	CTSC	Dipeptidyl peptidase 1	0.458	0.05	p < 0.001
	HSP90B1	Endoplasmin	2.289	0.223	p < 0.001
FGF2/VEGF/BMP4	APOA4	Apolipoprotein A-IV	1.934	0.179	p < 0.001
	GC	vitamin D-binding protein	2.630	0.255	p < 0.001
	C4A	Complement C4	3.837	0.383	p < 0.001
	CP	Ceruloplasmin	2.010	0.137	p < 0.001
	GNB2L1	Receptor of activated protein C kinase 1	0.397	0.042	p < 0.001

**Figure 6:**
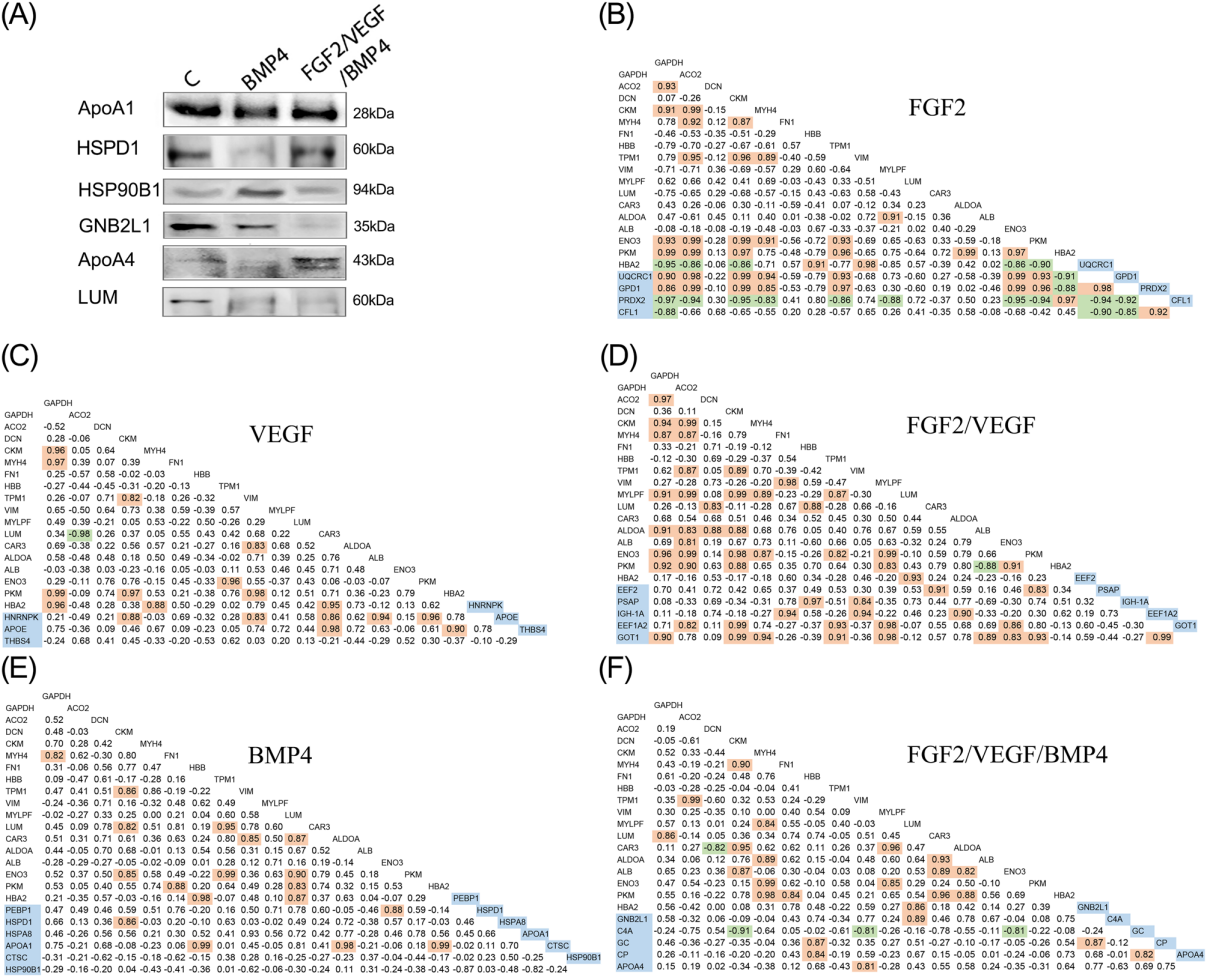
(**A**) Detection of differentially expressed proteins with specific antibodies. Equal amounts of total proteins isolated from the interface of each implant with the bone defect were transferred to nitrocellulose membranes for immunodetection of ApoA1, ApoA4, HSPD1, HSP90B1, GNB2L1 and LUM by western blotting. (**B**–**F**) A graphical representation of the Pearson correlation analysis. Pearson correlation matrices of the investigated groups for common (uncolored) and unique (blue colored) differentially expressed proteins involved in the response to stimulus GO slim class are represented. Red and green colors indicate a significant (p < 0.05) and high (± 0.8 to ± 1) positive and negative correlations, respectively, suggesting a very strong relationship between proteins.

HSP90B1 was one of the differentially expressed proteins with an increased abundance (~ 2.29 fold) in the BMP4 group while HSPD1 (0.3 fold) and APOA1 (0.35 fold) were detected with low abundances (Table 1). The data have also revealed a significantly increased abundance of APOA4 (1.93 fold) in the FGF2/VEGF/BMP4. Western blot analysis confirmed the altered protein abundances in these groups with an enhanced healing process of the bone defect when compared to control (Fig. 6A). Interestingly, supporting our data these two components of HDL, APOA1 and APOA4, were previously detected down-regulated in the serum of patients with atrophic fracture^22^. Besides APOA4, vitamin D-binding protein (GC), ceruloplasmin (CP) and complement C4a (C4A) were found in our analysis to have significantly increased abundance in the FGF2/ VEGF/ BMP4 group than in control (Table 1). Receptor of activated protein C kinase 1 was the only unique differentially expressed protein of FGF2/ VEGF/ BMP4 group detected with a significant lower abundance (0.397 fold) than control and confirmed by western blot (Fig. 6A).

### Bioinformatics analysis of differentially expressed proteins

To examine the collinearity between the proteins involved in *response to stimulus* that correspond to both common and unique categories of differentially expressed proteins, Pearson correlation matrices were performed. Highly strong significant (± 0.85 to ± 0.98, *p* < 0.05) correlations were detected between all unique differentially expressed proteins of FGF group (Fig. 6B). In contrast, VEGF group had no strong correlations for this category of proteins (Fig. 6C). However, numerous positive strong correlations were detected between unique and common differentially expressed proteins of the VEGF group. In the FGF2/ VEGF group (Fig. 6D) we detected one significant positive correlation in the unique differentially expressed proteins category that was established between cytoplasmic isoenzyme of aspartate aminotransferase (GOT1) and elongation factor 1-alpha 2 (EEF1A2).

In the BMP4 group, no significant correlations were detected between the unique differentially expressed proteins (Fig. 6E), even though HSP90B1 showed strong correlations with APOA1 (*r* = 0.82, *p* = 0.18) and phosphatidylethanolamine—binding protein 1 (PEBP, *r* = 0.87, *p* = 0.128). Of this category of proteins, only ApoA1 and HSPD1 showed significant correlations with common differentially expressed proteins. FGF2/VEGF/BMP4 group showed two significant correlations between the unique differentially expressed proteins (Fig. 6F). Therefore, CP and GNB2L1 correlated positively with GC (*r* = 0.82; *r* = 0.87, *p* < 0.05). Several strong significant correlations have also been detected between the unique and common differentially expressed proteins. Among these, APOA4 and GNB2L1 showed significant positive correlations with vimentin (VIM; *r* = 0.81, *p* = 0.026) and LUM (*r* = 0.89, *p* = 0.008), respectively.

### Interaction networks construction for correlated proteins

**Figure 7:**
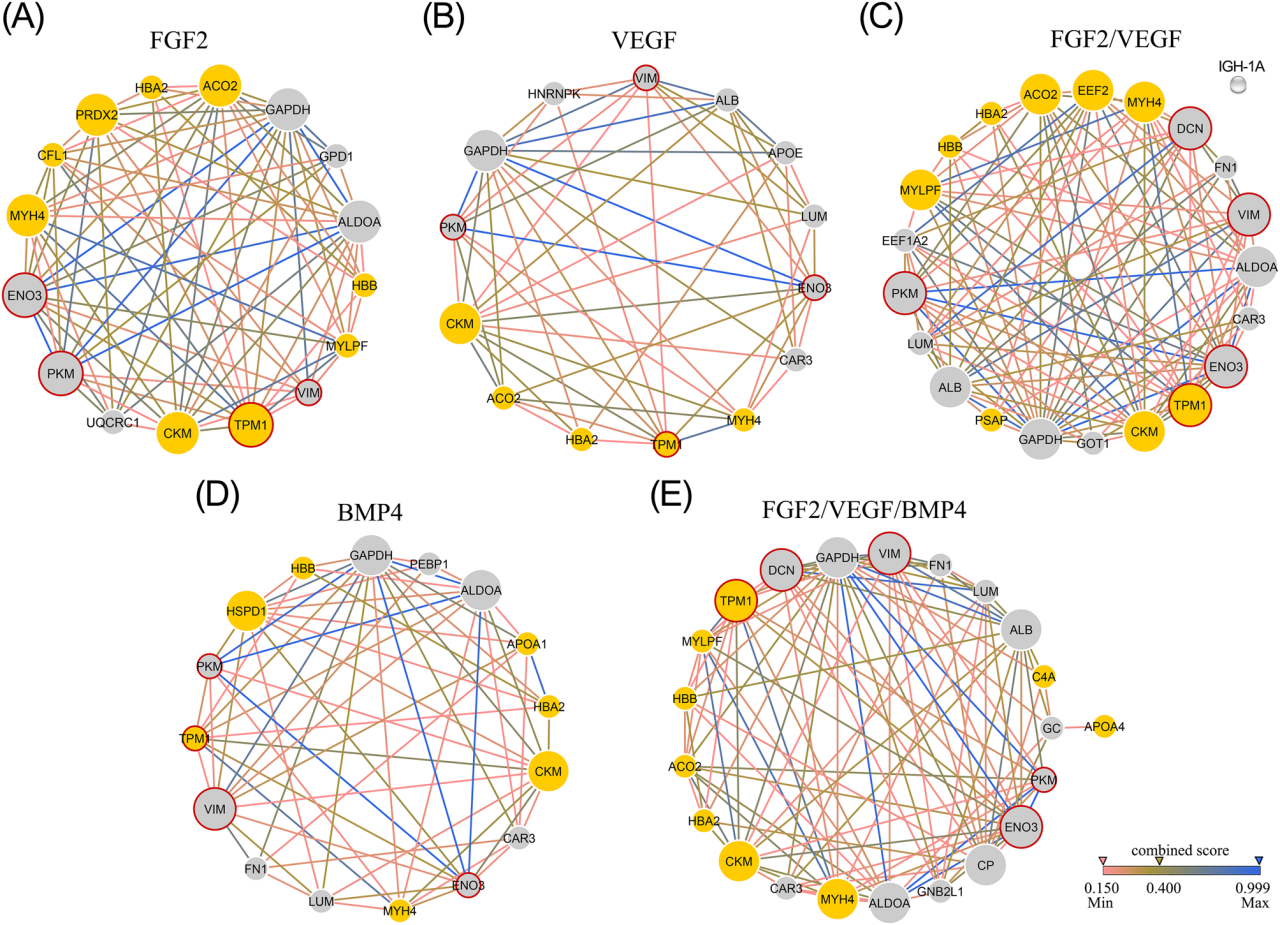
STRING analysis of correlated proteins (**A**–**E**)*.* Based on confidence interactions, the investigated groups show possible networks of proteins: colored nodes represent proteins and edges represent interactions between two correlated proteins. Grey nodes indicate participating proteins to the *response to chemical* and colored nodes with red margin show the proteins involved in *wound healing*. Big nodes represent proteins with interaction degree ≥ 10.

Next, the STRING database version 10.5 was used to reveal whether correlated proteins of investigated groups could generate possible protein networks. Based on confidence prediction of the interaction between proteins, a protein network was built for each group visualized using Cytoscape 3.5.1 (Fig. 7A–E).

All networks had significantly more interactions than expected and a clustering coefficient between 0.6 (FGF2) and 0.717 (FGF2/VEGF/BMP4), meaning that the proteins are linked as a macromolecular functional unit. However, based on the existing registered databases, Ig gamma-2b chain C region protein (IGH-1A) found in the FGF/VEGF group did not establish any interaction with the other proteins (Fig. 7C) even though from our results assessed by bioinformatics analysis, IGH-1A (AQR med 0.38) presented a significant correlation (*r* ≤ 0.9, *p* < 0.01, Fig. 7C) with fibronectin (FN1; AQR med 0.4), VIM (AQR med 0.36) and fructose-bisphosphate aldolase A (ALDOA; AQR med 0.14).

Interactions with the highest combined score (over 0.9) were noticed between pyruvate kinase PKM (PKM), beta enolase (ENO3) and glyceraldehyde-3-phosphate dehydrogenase (GAPDH) in all groups while APOA1 (AQR med 0.35) and Hemoglobin subunit beta-1 (HBB; AQR med 0.27) interaction was present only for BMP4 group (Fig. 7D). ALDOA was also connected with PKM, ENO3 and GAPDH in all groups excepting VEGF condition. LUM and decorin (DCN), two proteoglycans involved in collagen fibrillogenesis, are shown to have a strong interaction (combined score of 0.899) in FGF2/VEGF (Fig. 7C) and FGF2/VEGF/BMP4 (Fig. 7E) groups and both proteins are connected through medium interactions with GAPDH.

All networks had potential hub proteins selected on the basis of the high values of the betweenness centrality parameter and an interaction degree ≥ 10. Of these, GAPDH, ENO3, creatine kinase M-type (CKM) and albumin (ALB) were common to all groups. In addition, STRING analysis showed that correlated proteins of each investigated group were associated with several signaling pathways and part of them were common for all groups including *response to chemical* (GO: 0042221, FDR < 0.001), *wound healing* (GO: 0042060, FDR < 0.001) and *response to stress* (GO: 0006950, FDR < 0.001).

## Discussion

The combination of growth factors and biomaterial scaffolds represents a promising approach to provide a local improved regenerative treatment of bone defects and wound healing. In this study, we showed that a coated titanium implant with FGF2, VEGF and BMP4 incorporated into PEG speeded up the healing process in a rat tibial defect model. The choice of PEG was decided by its ability to cover the immunogenicity of biomolecules and to offer protein stability and protection from proteolytic degradation^23^.

Our in vitro data demonstrated that the coatings produce no cytotoxic effects and promote adequate adherence for cell proliferation. In vivo model confirmed through X-ray images, and histological and histomorphometric analysis that FGF2/VEGF/BMP4 group had an advanced remodeling phase at 6 weeks after surgery in comparison with the other examined implants that had similar growth factors, individually deposited or in binary combinations. An enhanced healing process was also detected for BMP4 and control groups. In contrast, VEGF group that was the most heterogenic group, presented a delay of the bone healing. The explanation for this might be related to an abnormal ossification and bone remodeling generated by exogenous VEGF. In this regard, in VEGF group were detected proteins of the cartilage extracellular matrix, such as prolargin (AQR med 8.6, p < 0.001) and aggrecan core protein (AQR med 5.13, p < 0.001) with higher abundances than control at 6 weeks after surgery. It has been reported that a concentration of VEGF beyond physiological needs fails to enhance intramembranous bone formation in vivo and osteoblast differentiation and mineralization in vitro ^24^*.* A similar result regarding a poor healing was also obtained for implants with FGF2. Our results are in line with the studies of Nakajima et al.^20^ and Bland et al.^21^ concerning the capacity of exogenous FGF2 to enlarge the cartilaginous calluses, but not to induce a rapid healing in closed fractures of rat and rabbit model, respectively.

The quantitative proteomic analysis performed in this study led to the identification of common but also unique proteins actively involved in the new tissue regeneration located at the interface of each implant with the bone defect. Therefore, we detected altered abundances for HSP90B1 and HSPD1 in the BMP4 group that had a second most enhanced healing process after FGF2/VEGF/BMP4 group. According to previous studies, HSP90B1 was documented to be a molecular chaperone in the regulation of signaling pathways in osteoblastogenesis including the upregulation of glucocorticoid receptor^25^ involved in the suppression of osteoblast differentiation^26^ while HSPD1 was showed to enhance the osteoblast survival and bone formation by inhibiting the effect of glucocorticoid treatment^27,28^. In our model, when the BMP4 was associated with FGF2 and VEGF, the HSPs did not pass the threshold set for proteomic quantification analysis suggesting that the 3D hierarchical structures of titanium implant generated a new balance between the proteins involved in tissue repair.

Previous reports showed that the deglycosylated form of GC can act as a macrophage activating factor and stimulate osteoclasts activity and bone resorption^29,30^, while high doses of GC may have osteoinductive effect^31^. In the serum of patients with cross-shaft (diaphyseal) long bone fracture an upward trend in GC concentration was observed over the 6 weeks observation period^32^. In our experimental model, the high abundance of GC detected in the FGF2/VEGF/BMP4 group after 6 weeks was significantly and strong correlated with ceruloplasmin, the primary copper transporting protein involved in the wound healing process. This result is supported by both in vitro as well as in vivo published models, in which copper facilitates angiogenesis, from early events to stabilization of newly formed blood vessels^33^.

The titanium-induced osseointegration was correlated with complement system through the upregulated C5a Receptor-1, associated with osteogenic differentiation, and the decreased C3 levels around the titanium implant, thought to be part of the bone resorption pathways suppression^34^. Here, in the described experimental model, only complement anaphylatoxin C4a had a significant increased abundance in the tissue situated between the implant and the new bone of the FGF2/ VEGF/ BMP4 group. Interestingly, C4a induces stress fiber formation and increases dose dependently endothelial permeability as shown by Wang et al.^35^. Having this insight, we suggest that the GC, CP and C4A may contribute to the rapid healing of the bone defect detected in the FGF2/VEGF/BMP4 group.

Receptor of activated protein C kinase 1 is a multifunctional protein involved in generation of osteoclasts by selective activation of p38 MAPK in osteoclast precursors^36^ and cell migration, cell adhesion and cell spreading^37^. In the FGF2/ VEGF/ BMP4 group that had the highest level of bone healing, it was positively correlated with the proteoglycan lumican. Interestingly, lumican was detected to be differentially expressed with a low abundance in all investigated groups compared to control. This down-regulation is surprising since previous studies showed that lumican promotes normal wound healing^38^ and its expression increases in osteoblasts during the matrix maturation^39^. The decreased abundances of lumican and receptor of activated protein C kinase 1 are difficult to interpret, however, these may be related with their temporal expression in the healing process. These uniquely differentially expressed proteins detected in FGF2/ VEGF/ BMP4 are also involved in the innate immune response that might be induced by the presence of the implant itself. Therefore, in a subsequent comparative study, it must be carefully determined whether these proteins could have an altered protein level generated by the capacity of the implant to produce a foreign body reaction in the remodeling tissue stage.

Consequently, the data of this study brought novel elements related to the improvement of bone healing process recovery after traumatic events. The experimental data, registered recently as patent application, showed that the titanium implant with bioactive designed surface containing optimized proportion of FGF2, VEGF and BMP4 stimulate bone healing in a rat tibia model. The proteomics results of this study revealed distinct quantitative proteomic profiles of the newly regenerated tissue located at implant: new bone interface from each experimental group with common and unique proteins that are differentially expressed. Further investigations are required to truly understand the roles and how the interactions of differentially expressed proteins exert their impact on the complex bone healing process.

## Methods

### Materials

Reagents were purchased from Millipore Sigma (CA, USA) and Sigma-Aldrich (MO, USA). Antibodies for immunocytochemistry and Western blot were purchased from Thermo Fisher Scientific (IL, USA). Reagents for mass spectrometry analysis were of specific grade.

### Fabrication of the implants

Sterilized titanium plates (22 × 2.5 × 0.6 mm) bent at the same curvature with that of the rat tibia bone were used as mechanical support for implants fabrication. Holes with 1.5 mm diameter were made in each plate and fixed with screws on the tibia bone. The titanium implants were successively cleaned into an ultrasonic bath with acetone, ethanol and deionized water for 15 min each and dried in a jet of high purity nitrogen. The best adhesion and stability of the PEG (6000 Da) based coatings on the titanium surface were determined at a volume ratio of 1:4 for the UV purified deionized H_2_O to C_2_H_5_-OH and was kept constant for all solutions. Five different sterile solutions used to coat the titanium implants were prepared as follows: Solution A: VEGF (10 ng/mL); Solution B: FGF2 (2.5 ng/mL); Solution C: VEGF (10 ng/mL) and FGF2 (2.5 ng/mL); Solution D: 4% (w/v) aqueous solution of P(3HB-3HV)-PEG- BMP4 spheres; Solution E: 4% (w/v) aqueous solution of P(3HB-3HV)-PEG-BMP4 spheres with VEGF (10 ng/mL) and FGF2 (2.5 ng/mL). P(3HB-3HV)-PEG spheres were prepared using a previously reported method^40^. Titanium implants were prepared by dip coating method^41^ at 10 mm/min withdrawal speed and subsequently heated at 37 °C for 30 min to promote the uniform evaporation of the solvents, the film densification and increased adhesion to the titanium surface. The specific liberation of VEGF during time was measured by in vitro test for implant with 10 ng/ml VEGF embedded into the matrix. The release into the culture medium of the VEGF was determined using the *DuoSet Elisa development system Human VEGF* (R&D System). The amount detected was between 0.6—5.13 pg/ ml at 3 h, 19 h, 29 h, 48 h. All steps were performed in a clean room environment to prevent pathogens contaminations. In the in vitro experiments, titanium discs with a diameter of 8 mm and 2 mm thick that have been prepared in the same manner as in vivo implants.

### Culture of mesenchymal stem cells and endothelial cells

Human mesenchymal stem cells (hMSCs) were isolated and immunophenotyped as previously published^42^. In order to perform cell adhesion and proliferation assays, 5000 cells/cm^2^ hMSCs or 12,500 cells/cm^2^ ECs, line EA.hy926^43^ were seeded in complete DMEM low-glucose media with 10% fetal bovine serum (EuroCloneSpA, Italy) and antibiotics (100U/l penicillin, 100U/l streptomycin, 50U/l neomycin) on the titanium discs coated with different combination of growth factors placed in 24-well plates (Corning, NY, USA) at 37 °C and 5% CO_2._

### Immunofluorescence microscopy of cells adherent to the coated implants

After 72 h, cells fixed with 4% paraformaldehyde (10 min) and permeabilized with 0.2% TritonX-100 (3 min) were subsequently incubated (30 min) with primary anti-vinculin antibody. After PBS washing, Alexa Fluor 594-conjugated secondary antibody was added (1:400) together with Alexa Fluor 488 labeled Phalloidin (1:100) for 20 min to detect F-actin filaments. Finally, specimens were incubated with a 1:10,000 Hoechst solution for nuclei staining (1 min) followed by thorough PBS wash and mounting in Prolong Gold Antifade Reagent. Samples were scanned with TissueFAXS iPlus system (Tissue Gnostics, Austria) at 20 × magnification.

### In vivo experimental design

Eighteen adult male Wistar rats (255 ± 60 g) housed separately under controlled conditions were randomly divided into six groups of three animals each: the control group (C) received uncoated implants, three groups received individually coated implants with FGF2, VEGF and BMP4 and other two groups were implanted with the multi-component implants (FGF2/ VEGF and FGF2/ VEGF/ BMP4). The animals were euthanized after 6 weeks and the tibiae with the bone defect were harvested for histological, mass spectrometry and western blot analyzes. Animal experiments were conducted according to the National Institutes of Health guide for the care and use of Laboratory animals (NIH Publications No. 8023, revised 1978), EU Directive 2010/63/EU for animal experiments and Romanian Law no. 471/2002. The protocol of the animal surgeries was approved by the Ethics Commission within the Scientific Committee of the Institute of Cellular Biology and Pathology “N. Simionescu” (accredited by the Order No. 789 of February 21th 2008, according to the national Law No. 206 of May 27th 2004).

### Surgical procedure

During surgery, the animals were put under general anesthesia induced with i.p. injection of 0.35 mg/kg of medetomidine (Domitor, Orion Corp., Finland) and 65 mg/kg ketamine (Ketaminol 10, Intervet International BV, Holland). The right rear leg of the rats was shaved, disinfected with ethyl alcohol (75%) and a lateral skin incision was made. When the tibia diaphysis was exposed, a lavage was performed with a sterile saline solution and 1 mm bone defect was generated between the central and proximal third of the anterolateral face of the tibia crest using a 0.8 mm drill. The implants were fixed with 3–4 titanium screws and the skin closed with 5–0 non-absorbable silk sutures (Ethicon, Inc., USA). The length (22 mm) of the titanium plate was selected due to the advantage generated by fixing the ends of the implant to the epiphysis which ensured a good stability. The animals received 4 mg/kg clindamycin antibiotic (Stada Hemofarm S.R.L., Romania) and 20 mg/kg tramadol retard (Krka D.D. Novo Mesto, Slovenia) for analgesia. The treatment was maintained in their drinking water for three days after surgery. The recovery of animals was monitored daily.

### Radiographic evaluation

Anterior–posterior and mediolateral biplanar radiographs (52 kV/2.5 mAs) were performed at 2 and 4 weeks after surgery using BuckyDiagnost System (Philips, Germany) under light anesthesia. Hind limbs of the animals were radiographed positioned in dorsal and lateral recumbency.

### Histology and histomorphometry of harvested tibiae

The harvested tibiae were evaluated using the STEMI 200-c stereomicroscope (Carl Zeiss, Germany). The bone pieces were fixed in 10% formalin solution, decalcified in 10% trichloroacetic acid for 3 days, dehydrated in ethyl alcohol, cleared in xylene and embedded in paraffin. Serial sections (7 μm) were cut using a Leica RM2245 microtome (Leica Biosystems Nussloch GbH., Germany) and stained with Movat pentachrome to differentiate mature from immature bone. This procedure was specific for: bone collagen fibers (yellow-violet), cartilage tissue (blue-green), collagen (yellow), elastic fibers and nuclei (dark brown) new osteoid (red). For semiquantitative histomorphometric analysis, the tissue sections (2 sections/ defect; 6 sections/ group) were automatically scanned by the TissueFAXS iPlus system (TissueGnostics, Austria) based on a ZEISS Axio Imager Z1 microscope, using 20 × magnification. The defect areas containing woven bone and cartilage tissue were quantified with ImageJ image processing software. A manual color threshold was applied and the measured areas were reported as percent of the defect area.

### Liquid chromatography–tandem mass spectrometric (LC–MS/MS) analysis

An equal amount of tissue (5 mg) was harvested from the proximal vicinity of the repaired bone defect of each animal. The samples were suitably processed for nano-chromatography and mass spectrometric analysis (LC–MS/MS), as mentioned previously^44^. The experiments were carried out using the EASY n-LC II system coupled to the LTQ OrbitrapVelos Pro mass spectrometer (Thermo Scientific, CA, USA). The same amount of peptides mixtures (1 µg) was separated and eluted using a 90 min 3–25% solvent B (99.9% acetonitrile and 0.1% formic acid) over solvent A (99.9% water and 0.1% formic acid) gradient, at 300 nL/min. The MS was operated in a Top12 data-dependent acquisition. Proteome Discoverer 1.4 (Thermo Scientific) and Mascot 2.5.1 (Matrix Science, UK) were used for protein inference in UniProtKB/ SwissProt fasta database. Variable (methionine oxidation, asparagine and glutamine deamidation) and fixed (cysteine carbamidomethylation) modifications were taken into account. The peptide false discovery rate was set below 0.05. Label-free relative quantification analysis was performed with Sieve 2.1 (Thermo Scientific) and a spectral abundance alteration of at least 1.5-fold (over the control group) corroborated with a *p-value* < 0.05 was considered a significant protein level variation. Precursor ion intensities were normalized using the total ion current algorithm. Proteins with more than 3 missing average quantification ratios (AQR) and those with a median AQR between 0.67 and 1.5 were excluded from the analysis. Protein Center 3.41 (Thermo Scientific) was used for disparities evaluation of the identified proteins and gene ontology (GO) annotation. InteractiVenn^45^ was utilized for protein sets comparison. To detect and visualize the possible interaction networks of differentially abundant proteins, we used STRING (Search Tool for the Retrieval of Interacting Genes) database^46^ and the Cytoscape3.5.1 software^47^.

### Western blot assay

Proteins (40 μg/lane) separated by SDS-PAGE and transferred to nitrocellulose membranes were exposed to specific primary antibodies against apolipoprotein A-I (APOA1), apolipoprotein A-IV (APOA4), 60 kDa heat shock protein, mitochondrial (HSPD1), endoplasmin (HSP90B1), receptor of activated protein C kinase 1(GNB2L1) and lumican (LUM), diluted 1:1000 in TBS with 1% BSA followed by the appropriate horseradish peroxidase (HRP)-conjugated secondary antibody (diluted 1:3000). The immune complexes were detected by enhanced chemiluminescence.

### Statistical analysis

Histomorphometric data are presented as box plots showing medians, the 25–75% percentiles and whiskers that represent the minimum and maximum values. For two groups’ comparison, unpaired two-tailed Student’s* t* test was used and p values less than 0.05 were considered significant. Pearson correlation analysis was used to calculate the correlation coefficient between commonly and uniquely differentially expressed proteins identified by LC–MS analysis in all groups. The significance was set at p < 0.05. Statistical analysis was carried out by using GraphPad Prism Version 5.00 (San Diego, USA).

## Supplementary information

Supplementary Information.

## Data Availability

The mass spectrometry datasets generated during the current study are available in the ProteomeXchange Consortium via the PRIDE^48^ partner repository, with the dataset identifier PXD016135 and 10.6019/PXD016135.
