# Supplementary material for: Proteomics of regenerated tissue in response to a titanium implant with a bioactive surface in a rat tibial defect model
Source: Sci Rep. 2020 Oct 28;10:18493. doi: 10.1038/s41598-020-75527-2 (PMC7595204; doi:10.1038/s41598-020-75527-2)
Supplement: Supplementary file 1 — Supplementary Information. [file 41598_2020_75527_MOESM1_ESM.pdf]

Supplementary information

Proteomics of regenerated tissue in response to a titanium implant with a bioactive surface in a rat tibial defect model

Raluca M. Boteanu [1], Viorel I. Suica[1], Luminita Ivan[1], Florentina Safciuc[1], Elena Uyy[1], Emanuel Dragan[1], Sorin M. Croitoru[2], Valentina Grumezescu[3], Marioara Chiritoiu[4], Livia E. Sima[4], Constantin Vlagioiu[5], Gabriel Socol[3*], Felicia Antohe[1*]

[1]Institute of Cellular Biology and Pathology “N. Simionescu” of the Romanian Academy, Bucharest, Romania

[2]Faculty of Engineering and Management of Technological Systems, Politehnica University of Bucharest, Romania

[3]National Institute for Lasers, Plasma and Radiation Physics, Bucharest, Romania

[4]Institute of Biochemistry of the Romanian Academy, Bucharest, Romania

[5] Faculty of Veterinary Medicine, University of Agronomic Sciences and Veterinary Medicine of Bucharest, Romania

[6]Faculty of Applied Chemistry and Materials Science, Politehnica University of Bucharest, Bucharest, Romania

Corresponding authors: Felicia ANTOHE, PhD
Institute of Cellular Biology and Pathology “N. Simionescu”
8, B.P. Hasdeu Street, PO Box 35-14, 050568
Bucharest, Romania, Phone: +4021 319 45 18
Email: felicia.antohe@icbp.ro
and
Gabriel SOCOL, PhD
National Institute for Lasers, Plasma and Radiation Physics
409 Atomistilor Street, P.O. Box MG-54 RO-77125
Magurele, Bucharest, Romania, Phone: +4021 457 44 91
Email: gabriel.socol@inflpr.ro

Cell Proliferation Assay

The cytocompatibility of the polymer scaffolds containing growth factors was evaluated using the MTS assay (Promega, WI, USA) for hMSC or MTT Sigma-Aldrich (MO, USA) for EC according to manufacturer specifications. On the third day after hMSC seeding, the culture medium was removed and a pre-warmed solution of MTS in complete media was carefully added to the 24-wells and maintained for 1 h. Then, the supernatants were divided between the wells of a 96-well plate in duplicates and the absorbance was recorded at 450 nm using a Mithras plate reader (Berthold, Germany). Data represents averages +/- SEM of two independent experiments (n=6). In parallel experiments, at 72h post –seeding confluent endothelial cells were treated with MTT reagents and the absorbance at 570 nm was recorded. The background absorbance measured at 690 nm was systematically subtracted from each well.

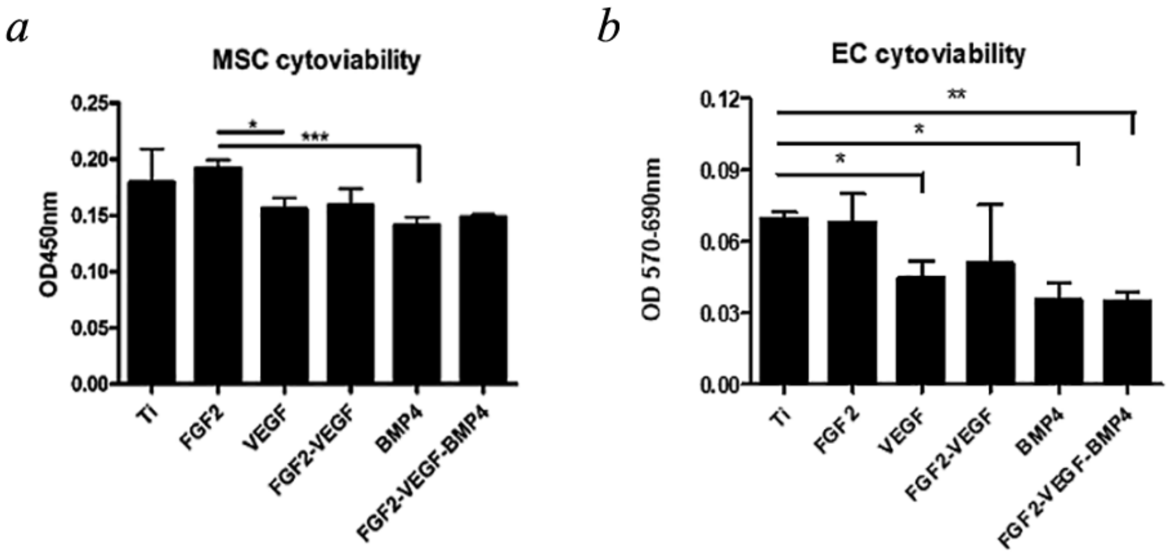

Figure S1: Cytoviability of mesenchymal stem cells (*a*) and endothelial cells (*b*) after 72h growth onto tested biomaterials. (*a*) Human MSCs proliferation was increased by the FGF2 released from the polymeric matrix as compared to titanium control, as well as VEGF, FGF2/VEGF, BMP4 or FGF2/VEGF/BMP4 combination. (*b*) Similar behavior could be observed for endothelial cells that showed increased proliferation while growing on titanium and in the presence of FGF2 and FGF2/VEGF coatings. Ti: titanium; FGF2: fibroblast growth factor 2; VEGF: vascular endothelial cell growth factor; BMP4: bone marrow protein 4. Unpaired two-tailed Student's *t* test: *p<0.05; **p<0.01, ***p<0.001.

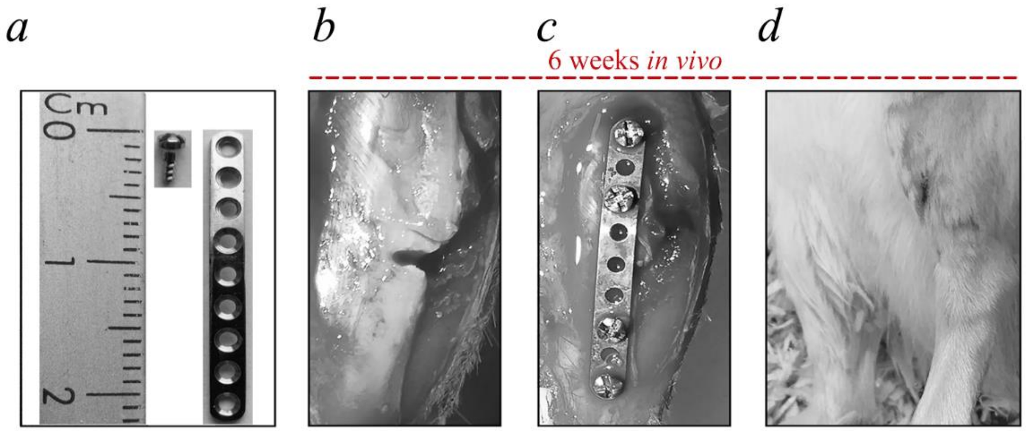

Figure S2: Rat animal model of tibia injury (a) Representative image of titanium plate and screw used in this study; (b) Initial intraoperative view of the tibial bone defect; (c) Titanium composite implant fixed on top of bone lesion with 4 titanium screws; (d) Intermediary view of healing wound after 2 weeks from surgical procedure.

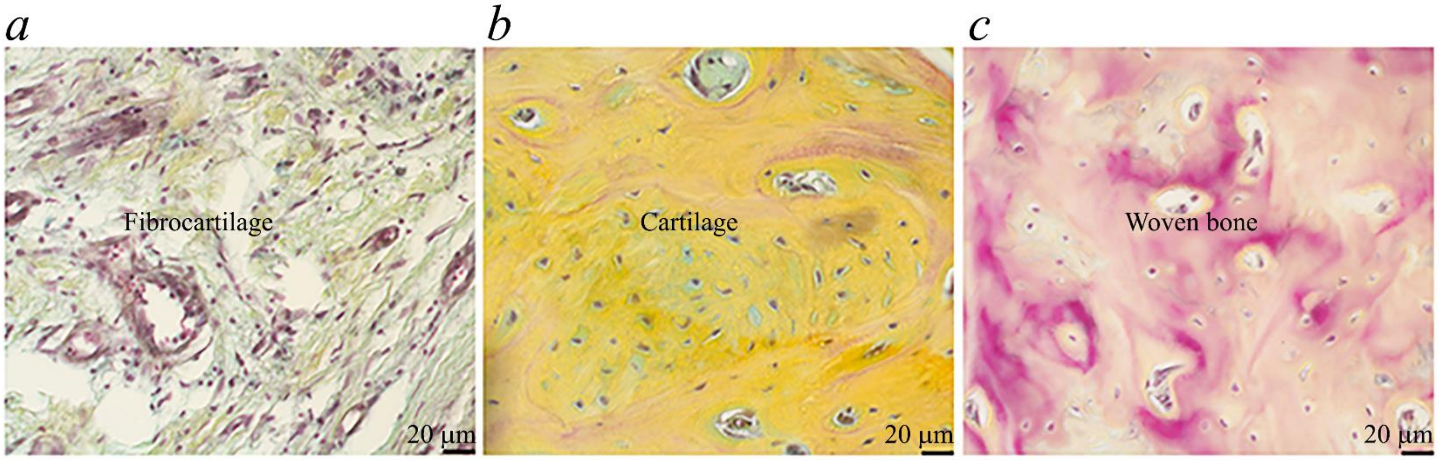

Figure S3: Histological evaluation with Movat's pentachrome staining of VEGF animals (a - c) showing the large heterogeneity of the healing stages in this group. Six weeks post-implantation, VEGF mice presented at defect site either a soft callus (a) or a hard callus that was found to be in the cartilage mineralization phase (b) or in the bone formation phase combined with the remodeling phase (c).

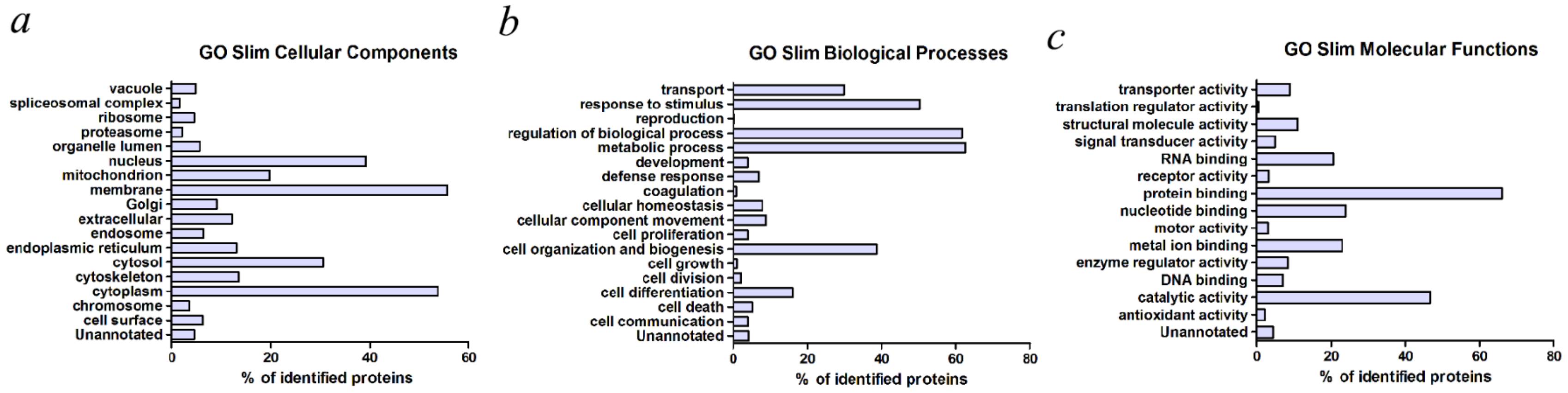

Figure S4: Gene Ontology (GO) slim analysis was performed on merged list of all identified proteins (1614). According to GO slim cellular components analysis, proteins were classified into 18 cellular component groups, of which two were dominant namely *cytoplasm* and *membrane* that account over 50% of identified proteins (Figure S2*a*). As shown in Figure S2*b*, classification in terms of biological processes revealed a high percent of proteins involved in *metabolic process* (62.5%), *regulation of biologic process* (61.7%) and *response to stimulus* (50.3%). Functionally, most of the proteins were grouped in *protein binding* (66%) and *catalytic activity* (46.6%). Each GO slim classification domains presented *Unannotated* proteins.

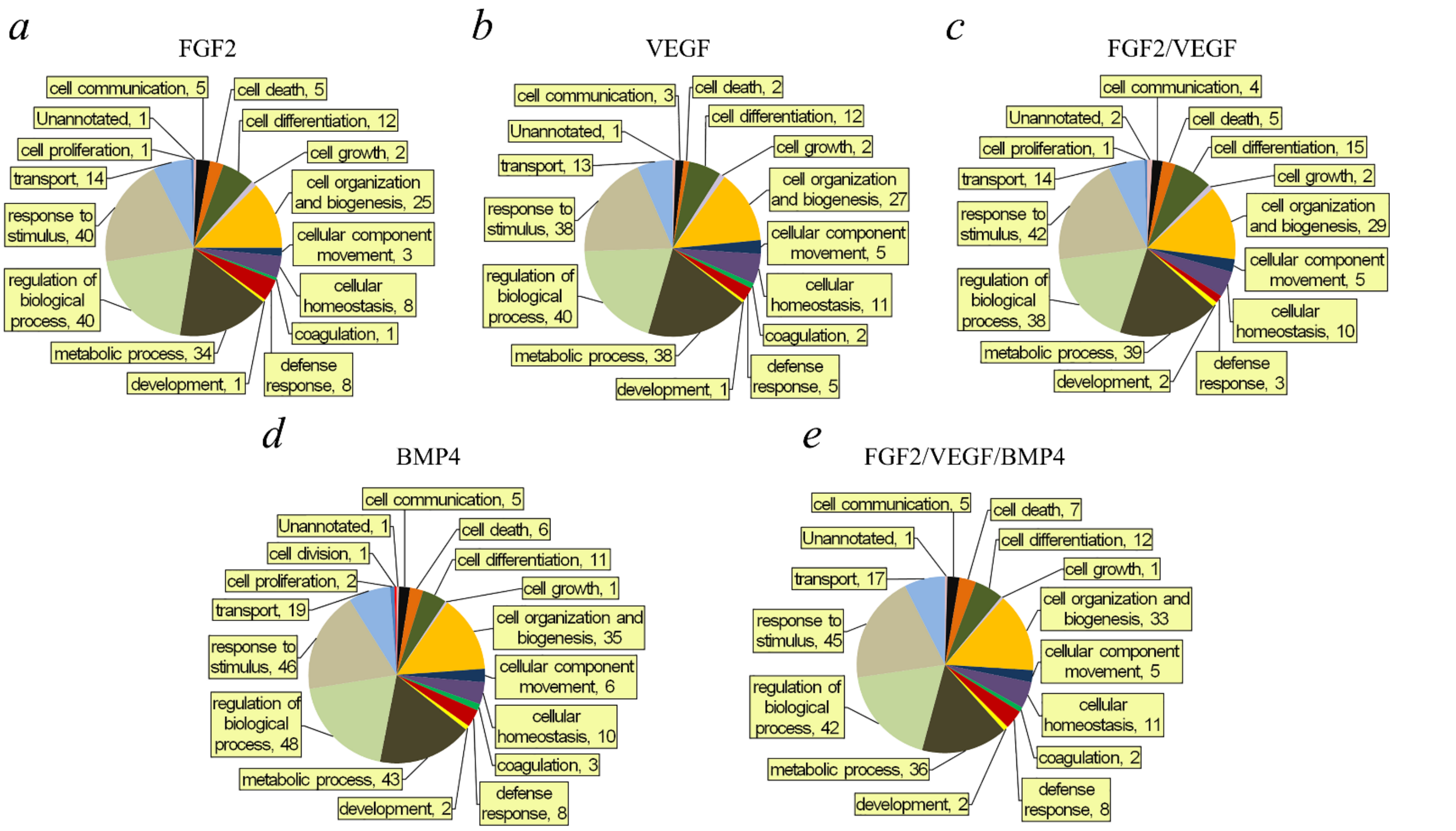

Figure S5. GO Slim biological processes categories of the differentially expressed proteins are shown in pie charts. For the FGF2, FGF2/ VEGF and FGF2/ VEGF/ BMP4 groups, *response to stimulus* clustered the 70%, 68% and 75% of differentially expressed proteins respectively, representing the dominant class. Instead, VEGF and BMP4 groups had *regulation of biological process* as main class, followed closely by the *response to stimulus* category. For all investigated groups, *response to stimulus*, *regulation of biological process* and *metabolic process* gathered the most proportion of the differentially expressed proteins.

Western blot assay Supplementary Information for Figure 6.

a)

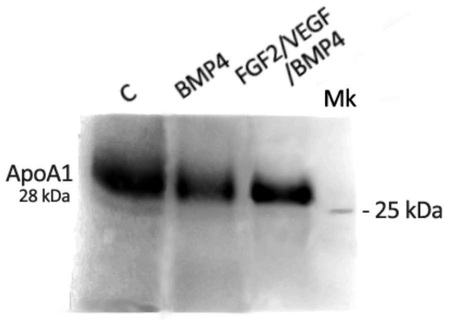

b)

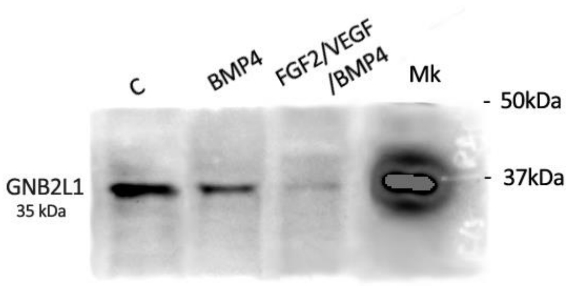

c)

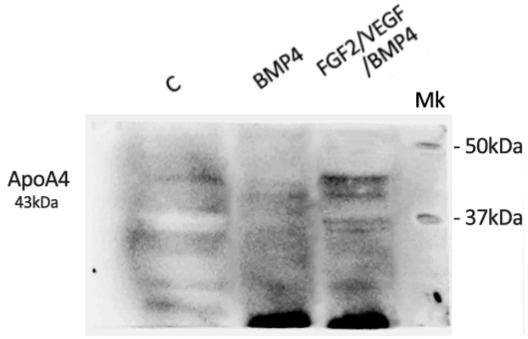

d)

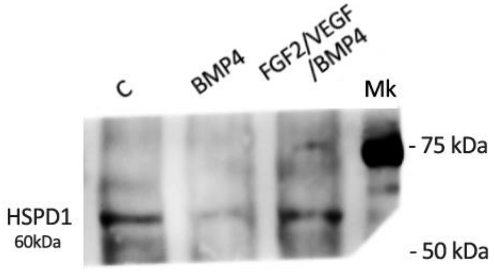

e)

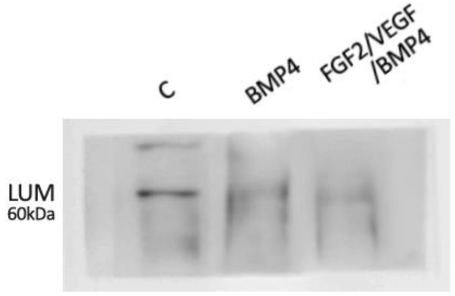

f)

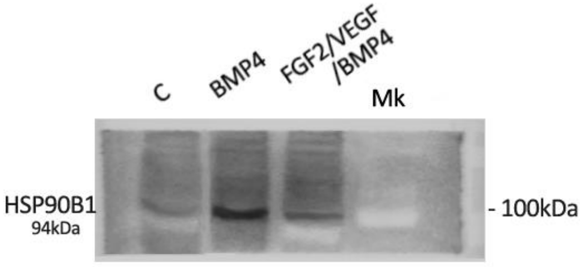

Figure S6. The full uncropped nitrocellulose Western Blotting images used to evidence the positive reaction for *a)* ApoA1, *b)* GNB2L1, *c)* ApoA4, *d)* HSPD1, *e)* LUM and *f)* HSP90B1 shown in Figure 6.

Supplementary Table
Table S1: List of all differentially expressed proteins

GO Slim Molecular Functions: antioxidant activity, catalytic activity, DNA binding, enzyme regulator activity, metal ion binding, motor activity, nucleotide binding, protein binding, receptor activity, RNA binding, signal transducer activity, structural molecule activity, transcription regulator activity, translation regulator activity, transporter activity

GO Slim Cellular Components: cell surface, chromosome, cytoplasm, cytoskeleton, cytosol, endoplasmic reticulum, endosome, extracellular, Golgi, membrane, mitochondrion, nucleus, organelle lumen, proteasome, ribosome, spliceosomal complex, vacuole

GO Slim Biological Processes: cell communication, cell death, cell differentiation, cell division, cell growth, cell organization and biogenesis, cell proliferation, cellular component movement, cellular homeostasis, coagulation, conjugation, defense response, development, metabolic process, regulation of biological process, reproduction, response to stimulus, transport

ProteinCenter — 113 Proteins in Merged_prot filtrate

Key	No	O	Cluster	Gene	AA	AS	Tax	Molecular Functions	Cellular Components	Biological Processes	TM	SP	AQR med	QSD med	QPV med
P63039	8		-	Hspd1	573		Rn				1		0.300	0.038	6.325
	60 kDa heat shock protein, mitochondrial														
P06761	31		-	Hspa5	654		Rn				0		0.486	0.047	2.480
	78 kDa glucose-regulated protein														
Q9ER34	42		-	Aco2	780		Rn				1		0.378	0.033	6.480
	Aconitate hydratase, mitochondrial														
P68136	38		-	Acta1	377		Rn				0		0.279	0.023	2.895
	Actin, alpha skeletal muscle														
P17475	18		-	Serpina1	411		Rn				1		1.732	0.165	1.331
	Alpha-1-antiproteinase														
P14046	28		-	A1i3,LOC297568	1477		Rn				2		2.293	0.163	6.415
	Alpha-1-inhibitor 3														
Q63041	34		-	Pzp	1500		Rn				2		1.814	0.081	5.595
	Alpha-1-macroglobulin														
P24090	14		-	Ahsg	352		Rn				0		0.554	0.077	7.810
	Alpha-2-HS-glycoprotein														
P04764	24		-	Eno1	434		Rn				1		0.228	0.023	8.920
	alpha-enolase														
Q07936	27		-	Anxa2	339		Rn				0		0.471	0.046	3.370
	Annexin A2														
P48037	25		-	Anxa6	673		Rn				0		0.476	0.052	3.630
	annexin A6														
P04639	6		-	Apoa1	259		Rn				0		0.351	0.091	0.000
	Apolipoprotein A-I														
P02651	8		-	Apoa4	391		Rn				0		1.934	0.179	3.522
	Apolipoprotein A-IV														

No: Number of Experimental Data; O: Outdated status; Cluster: Cluster name; Gene: Gene official symbol; AA: Length of protein sequence; AS: Alternative splicing; Tax: Taxonomy name; TM: TransMembrane domains; SP: Signal Peptide; AQR med: Average quantitation ratio; QSD med: Standard deviation for quantitation ratio; QPV med: P-Value for quantitation ratio

Key	No O	Cluster	Gene	AA	AS	Tax	Molecular Functions	Cellular Components	Biological Processes	TM	SP	AQR med	QSD med	QPV med
P02650	8<br>Apolipoprotein E	-	Apoe	312		Rn				0		0.164	0.021	3.020
P13221	9<br>Aspartate aminotransferase, cytoplasmic	-	Got1	413		Rn				0		0.399	0.033	1.740
P10719	26<br>ATP synthase subunit beta, mitochondrial	-	Atp5b	529		Rn				2		0.574	0.034	6.390
P15429	49<br>Beta-enolase	-	Eno3	434		Rn				1		0.296	0.023	3.110
P47853	34<br>biglycan	-	Bgn	369		Rn				0		0.269	0.024	8.290
P35565	6<br>Calnexin	-	Canx	591		Rn				1		1.774	0.235	2.640
P18418	33<br>Calreticulin	-	Calr	416		Rn				0		0.449	0.057	6.020
B0BNN3	20<br>carbonic anhydrase 1	-	Ca1,Car1	261		Rn				1		1.168	0.056	2.140
P27139	16<br>Carbonic anhydrase 2	-	Ca2,Car2	260		Rn				1		0.414	0.026	3.115
P14141	45<br>carbonic anhydrase 3	-	Car3	260		Rn				0		0.424	0.022	5.460
P13635	8<br>Ceruloplasmin	-	Cp	1059		Rn				1		2.010	0.137	5.340
P11442	13<br>Clathrin heavy chain 1	-	Cltc	1675		Rn				1		0.453	0.032	8.330
P45592	8<br>Cofilin-1	-	Cfl1	166		Rn				0		2.303	0.176	6.540
P02454	33<br>Collagen alpha-1(I) chain	-	Col1a1	1453		Rn				0		0.365	0.026	5.880
P02466	24<br>Collagen alpha-2(I) chain	-	Col1a2	1372		Rn				0		0.333	0.033	9.490
P01026	31<br>Complement C3	-	C3	1663		Rn				4		2.498	0.166	3.880
P08649	9<br>Complement C4	-	C4a	1737		Rn				4		3.837	0.383	5.950
P00564	49<br>Creatine kinase M-type	-	Ckm	381		Rn				0		0.210	0.010	9.900

Number of Experimental Data
Outdated status
Cluster name
Gene official symbol
Length of protein sequence
Alternative splicing
Taxonomy name
TransMembrane domains
Signal Peptide
Average quantitation ratio
Standard deviation for quantitation ratio
P-Value for quantitation ratio

Key	No	O	Cluster	Gene	AA	AS	Tax	Molecular Functions	Cellular Components	Biological Processes	TM	SP	AQR med	QSD med	QPV med
P09605	6		-	Ckmt2	419		Rn				0		3.737	0.352	1.275
	Creatine kinase S-type, mitochondrial														
Q68FY0	8		-	Uqcrc1	480		Rn				0		0.465	0.045	7.800
	Cytochrome b-c1 complex subunit 1, mitochondrial														
Q01129	44		-	Dcn	354		Rn				0		0.249	0.020	1.485
	decorin														
P48675	36		-	Des	469		Rn				0		0.244	0.019	5.355
	desmin														
Q62952	19		-	Dpysl3	570		Rn				0		1.608	0.104	1.410
	Dihydropyrimidinase-related protein 3														
P80067	6		-	Ctsc	462		Rn				0		0.458	0.050	2.995
	Dipeptidyl peptidase 1														
P62630	15		-	Eef1a1	462		Rn				1		0.173	0.015	4.950
	Elongation factor 1-alpha 1														
P62632	11		-	Eef1a2	463		Rn				1		0.356	0.020	5.890
	Elongation factor 1-alpha 2														
P05197	9		-	Eef2	858		Rn				0		0.526	0.021	6.660
	Elongation factor 2														
Q66HD0	7		-	Hsp90b1	804		Rn				1		2.289	0.223	2.420
	Endoplasmin														
Q9QX79	8		-	Fetub	378		Rn				0		1.784	0.248	5.070
	Fetuin-B														
P14480	14		-	Fgb	479		Rn				0		1.696	0.092	2.068
	Fibrinogen beta chain														
P50609	15		-	Fmod	376		Rn				0		0.518	0.039	1.810
	Fibromodulin														
P04937	47		-	Fn1	2477		Rn				1		0.221	0.010	9.900
	fibronectin														
D3ZHA0	11		-	Flnc	2726		Rn				1		0.149	0.025	2.980
	Filamin-C														
Q9WUH4	7		-	Fhl1	280		Rn				0		0.087	0.008	3.900
	Four and a half LIM domains protein 1														
P05065	46		-	Aldoa	364		Rn				0		0.146	0.007	9.900
	fructose-bisphosphate aldolase A														
P14408	6		-	Fh	507		Rn				0		0.344	0.049	7.269
	fumarate hydratase, mitochondrial														

Number of Experimental Data; Outdated status; Cluster name; Gene official symbol; Length of protein sequence; Alternative splicing; Taxonomy name; TransMembrane domains; Signal Peptide; Average quantitation ratio; Standard deviation for quantitation ratio; P-Value for quantitation ratio

Key	No	O	Cluster	Gene	AA	AS	Tax	Molecular Functions	Cellular Components	Biological Processes	TM	SP	AQR med	QSD med	QPV med
Q68FP1	29<br>Gelsolin		-	Gsn	780		Rn				0		0.410	0.036	4.480
Q6P6V0	19<br>glucose-6-phosphate isomerase		-	Gpi	558		Rn				3		0.592	0.069	1.120
P04797	46<br>glyceraldehyde-3-phosphate dehydrogenase		-	Gapdh,LOC685186,LOC108351137	333		Rn				0		0.126	0.008	9.900
O35077	9<br>Glycerol-3-phosphate dehydrogenase [NAD(+)], cytoplasmic		-	Gpd1	349		Rn				4		0.597	0.037	3.660
P09812	36<br>Glycogen phosphorylase, muscle form		-	Pygm	842		Rn				2		0.376	0.031	2.225
Q9WTT6	21<br>guanine deaminase		-	Gda	454		Rn				0		0.352	0.039	1.480
P63018	6<br>Heat shock cognate 71 kDa protein		-	Hspa8	646		Rn				0		0.306	0.030	4.970
P01946	45<br>Hemoglobin subunit alpha-1/2		-	Hba-a2,Hba1,Hba2	142		Rn				0		0.331	0.011	9.900
P02091	43<br>Hemoglobin subunit beta-1		-	Hbb	147		Rn				1		0.467	0.029	5.180
P20059	15<br>Hemopexin		-	Hpx	460		Rn				0		0.092	0.009	3.660
P61980	6<br>Heterogeneous nuclear ribonucleoprotein K		-	Hnrnpk	463		Rn				0		0.277	0.022	5.640
P20759	14<br>Ig gamma-1 chain C region		-	Ighg,Ighg1	326		Rn				0		1.146	0.078	3.776
P20760	17<br>Ig gamma-2A chain C region		-	LOC679045	322		Rn				0		2.022	0.106	1.380
P20761	10<br>Ig gamma-2b chain C region		-	Igh-1a,Igh-6	333		Rn				0		0.386	0.070	4.271
P04642	25<br>L-lactate dehydrogenase A chain		-	Ldha	332		Rn				1		0.246	0.028	9.430
P51886	44<br>Lumican		-	Lum	338		Rn				0		0.418	0.026	1.098

Number of Experimental Data; Outdated status; Cluster name; Gene official symbol; Length of protein sequence; Alternative splicing; Taxonomy name; TransMembrane domains; Signal Peptide; Average quantitation ratio; Standard deviation for quantitation ratio; P-Value for quantitation ratio

Key	No	O	Cluster	Gene	AA	AS	Tax	Molecular Functions	Cellular Components	Biological Processes	TM	SP	AQR med	QSD med	QPV med
Q6AYC4	6		-	Capg	349		Rn				0		0.439	0.028	3.102
	Macrophage-capping protein														
Q9QZ76	24		-	Mb	154		Rn				0		0.496	0.036	5.330
	Myoglobin														
P02600	48		-	Myl1	189		Rn				0		0.068	0.004	9.900
	Myosin light chain 1/3, skeletal muscle isoform														
Q64119	9		-	Myl6l	151		Rn				0		0.221	0.021	1.180
	Myosin light polypeptide 6														
P04466	47		-	Mylpf	169		Rn				0		0.122	0.007	9.900
	Myosin regulatory light chain 2, skeletal muscle isoform														
Q29RW1	47		-	Myh4	1939		Rn				0		0.132	0.003	9.900
	Myosin-4														
P04462	35		-	Myh8	257		Rn				0		0.207	0.021	8.960
	Myosin-8														
Q62812	23		-	LOC100911597,Myh9,Myh9l1	1961		Rn				1		0.468	0.053	7.210
	Myosin-9														
Q63518	23		-	Mybpc1	621		Rn				1		0.111	0.012	3.680
	Myosin-binding protein C, slow-type														
P62961	8		-	Ybx1	322		Rn				0		1.648	0.114	1.723
	Nuclease-sensitive element-binding protein 1														
P02625	36		-	Pvalb	110		Rn				0		0.363	0.024	5.550
	Parvalbumin alpha														
P10111	27		-	Ppia,LOC100360977	164		Rn				0		0.413	0.047	2.440
	peptidyl-prolyl cis-trans isomerase A														
P35704	8		-	Prdx2	198		Rn				1		1.942	0.173	3.500
	Peroxiredoxin-2														
P31044	6		-	Pebp1	187		Rn				0		0.293	0.040	5.785
	phosphatidylethanolamine-binding protein 1														
P38652	16		-	Pgm1	562		Rn				1		0.326	0.028	8.215
	Phosphoglucomutase-1														
P16617	42		-	Pgk1	417		Rn				1		0.335	0.025	1.105
	phosphoglycerate kinase 1														
P16290	26		-	Pgam2	253		Rn				0		0.546	0.037	6.965
	phosphoglycerate mutase 2														

Number of Experimental Data; Outdated status; Cluster name; Gene official symbol; Length of protein sequence; Alternative splicing; Taxonomy name; TransMembrane domains; Signal Peptide; Average quantitation ratio; Standard deviation for quantitation ratio; P-Value for quantitation ratio

Key	No	O	Protein name	Cluster	Gene	AA	AS	Tax	Molecular Functions	Cellular Components	Biological Processes	TM	SP	AQR med	QSD med	QPV med
Q01177	16		Plasminogen	-	Plg	812		Rn				0		2.292	0.281	1.006
P30427	35		plectin	-	Plec	4687		Rn				1		0.429	0.022	1.900
P48679	34		Prelamin-A/C	-	Lmna	665		Rn				0		0.426	0.031	1.718
Q9EQP5	42		prolargin	-	Prelp	377		Rn				0		0.158	0.011	2.687
P10960	9		Prosaposin	-	Psap	554		Rn				0		1.694	0.054	9.900
P04785	24		Protein disulfide-isomerase	-	P4hb	509		Rn				0		0.418	0.029	9.825
P11598	37		Protein disulfide-isomerase A3	-	Pdia3	505		Rn				1		0.466	0.035	1.120
P11980	42		Pyruvate kinase PKM	-	Pkm	531		Rn				0		0.323	0.017	5.550
P63245	8		Receptor of activated protein C kinase 1	-	Gnb2l1,Rack1,LOC100911540	317		Rn				0		0.397	0.042	0.000
Q64578	15		Sarcoplasmic/endoplasmic reticulum calcium ATPase 1	-	Atp2a1	994		Rn				10		0.183	0.020	2.370
Q9WVC0	6		Septin-7	-	Sept7	436		Rn				0		0.335	0.039	4.725
P05545	16		Serine protease inhibitor A3K	-	Serpina3k,Serpina3c	416		Rn				1		1.945	0.120	6.835
P05544	20		Serine protease inhibitor A3L	-	LOC299282	413		Rn				0		1.227	0.083	2.730
P12346	37		Serotransferrin	-	Tf	698		Rn				1		0.430	0.025	6.310
P29457	33		Serpin H1	-	Serpinh1	417		Rn				1		0.481	0.068	2.340
P02770	47		Serum albumin	-	Alb	608		Rn				0		0.035	0.002	9.900
P16086	10		Spectrin alpha chain, non-erythrocytic 1	-	Sptan1	2472		Rn				0		0.388	0.050	1.028

Number of Experimental Data; Outdated status; Cluster name; Gene official symbol; Length of protein sequence; Alternative splicing; Taxonomy name; TransMembrane domains; Signal Peptide; Average quantitation ratio; Standard deviation for quantitation ratio; P-Value for quantitation ratio

Key	No	O	Cluster	Gene	AA	AS	Tax	Molecular Functions	Cellular Components	Biological Processes	TM	SP	AQR med	QSD med	QPV med
P49744	6<br>Thrombospondin-4		-	Thbs4	980		Rn				0	⌇	2.449	0.341	1.444
P50137	22<br>Transketolase		-	Tkt	623		Rn				1		0.465	0.053	1.070
P02767	6<br>Transthyretin		-	Ttr	147		Rn				0	⌇	0.432	0.023	2.185
P48500	9<br>Triosephosphate isomerase		-	Tpi1,LOC100911515	249		Rn				0		0.340	0.011	2.750
P04692	47<br>Tropomyosin alpha-1 chain		-	Tpm1	284	✓	Rn				0		0.217	0.016	2.950
Q63610	14<br>Tropomyosin alpha-3 chain		-	Tpm3	248	✓	Rn				0		0.206	0.021	1.734
P09495	9<br>Tropomyosin alpha-4 chain		-	Tpm4	248		Rn				0		0.339	0.021	6.100
P58775	43<br>Tropomyosin beta chain		-	Tpm2	284	✓	Rn				0		0.180	0.013	1.350
P27768	35<br>Troponin I, fast skeletal muscle		-	Tnni2	182		Rn				0		0.110	0.008	3.570
P09739	18<br>Troponin T, fast skeletal muscle		-	Tnnt3	259	✓	Rn				0		1.154	0.060	2.259
P31000	45<br>Vimentin		-	Vim	466		Rn				0		0.359	0.022	9.900
P85972	11<br>Vinculin		-	Vcl	1066		Rn				0		0.533	0.034	2.940
P04276	9<br>vitamin D-binding protein		-	Gc	476		Rn				0	⌇	2.630	0.255	6.470
Q9Z2L0	18<br>voltage-dependent anion-selective channel protein 1		-	Vdac1	283		Rn				0		0.525	0.050	1.509

Number of Experimental Data; Outdated status; Cluster name; Gene official symbol; Length of protein sequence; Alternative splicing; Taxonomy name; TransMembrane domains; Signal Peptide; Average quantitation ratio; Standard deviation for quantitation ratio; P-Value for quantitation ratio
